# Hospitals under 50 Beds

**Published:** 1916-12-16

**Authors:** 


### Hospitals under 50 Beds.

**GROSVENOR HOSPITAL FOR WOMEN.**—The immediate requirement of this institution is money. The debt to the bank is now about £800, an amount never approached before, and the falling off of subscriptions and donations is necessitating the earnest consideration of the committee as to whether they will not be compelled soon to close some of the beds in the institution. Secretary, Mr. W. J. Davidson, Vincent Square, Westminster.

**PADDINGTON GREEN CHILDREN'S HOSPITAL.**—This hospital provides forty-six cots, has a large up-to-date out-patient department, and an invaluable Convalescent Home at Slough. The in-patients average over 700 a year, and the out-patient attendances nearly 50,000. The average ordinary yearly expenditure of the hospital and home exceeds the ordinary income by about £1,000. This deficit has usually been met out of legacies, but owing to the serious falling off during the past three years in this uncertain source of income on the one hand, and, since the war, the greatly increased cost of maintenance on the other, money has had to be borrowed from time to time from the hospital's bankers until there is now a heavy debt upon the hospital. Unless immediate financial help is forthcoming, the committee will be unable to carry on the work. Contributions will be thankfully received by the Chairman, Sir Douglas Owen, or the Secretary, Mr. W. H. Pearce, at the hospital.

**ROYAL WESTMINSTER OPHTHALMIC HOSPITAL.**—In common with other institutions, this hospital is being adversely affected by the continued increase in price of all commodities, which is causing grave anxiety to the committee of management as to the expenditure on maintenance, which calls for a constant and increasing flow of contributions. One hundred years ago the institution was founded by the great Duke of Wellington, and as in that far-off time it is to-day aiding in the treatment of soldiers suffering from diseases and injuries of the eye, and the treatment, in many instances, prevents blindness, than which there can hardly be a more terrible calamity. Upwards of 17,000 new out-patients, whose attendances amount to nearly 40,000, are relieved annually. The present year is the centenary of the charity, and special help is appealed for. Acting Secretary, Mr. P. W. Bennett, at the hospital, King William Street, Strand, W.C.

**ST. PETER'S HOSPITAL, COVENT GARDEN.**—The appeal this Christmas is for £800 to meet the most pressing liabilities for maintenance. There are, of course, many other objects for which funds are needed, but it is felt that this is not the time to ask. Secretary, Mr. J. H. Beattie.

**WESTERN OPHTHALMIC HOSPITAL, MARYLEBONE ROAD.**—Every year the Western Ophthalmic Hospital, Marylebone Road, W., bestows treatment upon about 12,000 patients, the sight of thousands of eyes is saved, and after careful examination some 6,000 pairs of spectacles are prescribed. The cost of the work is less than £2,000 a year, but the hospital has been so neglected by the charitable public for a long course of years that its accumulated deficits are now represented by a total indebtedness of nearly £4,000. The committee would be most grateful for support, and they feel that the hospital's needs must appeal to a large section of the populace, because the great majority at one time or another derive inestimable benefits from ophthalmic advice. Mr. H. W. Burleigh, Hon. Secretary.

